# Deep learning identification for citizen science surveillance of tiger mosquitoes

**DOI:** 10.1038/s41598-021-83657-4

**Published:** 2021-02-25

**Authors:** Balint Armin Pataki, Joan Garriga, Roger Eritja, John R. B. Palmer, Frederic Bartumeus, Istvan Csabai

**Affiliations:** 1 Department of Physics of Complex System, ELTE Eötvös Loránd University, Budapest, Hungary; 2 Centre d’Estudis Avançats de Blanes (CEAB-CSIC), 17300 Girona, Spain; 3 CREAF, 08193 Cerdanyola del Vallés, Spain; 4 Universitat Pompeu Fabra, 08005 Barcelona, Spain; 5 Institució Catalana de Recerca i Estudis Avançats (ICREA), 08010 Barcelona, Spain

**Keywords:** Ecology, Diseases

## Abstract

Global monitoring of disease vectors is undoubtedly becoming an urgent need as the human population rises and becomes increasingly mobile, international commercial exchanges increase, and climate change expands the habitats of many vector species. Traditional surveillance of mosquitoes, vectors of many diseases, relies on catches, which requires regular manual inspection and reporting, and dedicated personnel, making large-scale monitoring difficult and expensive. New approaches are solving the problem of scalability by relying on smartphones and the Internet to enable novel community-based and digital observatories, where people can upload pictures of mosquitoes whenever they encounter them. An example is the Mosquito Alert citizen science system, which includes a dedicated mobile phone app through which geotagged images are collected. This system provides a viable option for monitoring the spread of various mosquito species across the globe, although it is partly limited by the quality of the citizen scientists’ photos. To make the system useful for public health agencies, and to give feedback to the volunteering citizens, the submitted images are inspected and labeled by entomology experts. Although citizen-based data collection can greatly broaden disease-vector monitoring scales, manual inspection of each image is not an easily scalable option in the long run, and the system could be improved through automation. Based on Mosquito Alert’s curated database of expert-validated mosquito photos, we trained a deep learning model to find tiger mosquitoes (*Aedes albopictus*), a species that is responsible for spreading chikungunya, dengue, and Zika among other diseases. The highly accurate 0.96 area under the receiver operating characteristic curve score promises not only a helpful pre-selector for the expert validation process but also an automated classifier giving quick feedback to the app participants, which may help to keep them motivated. In the paper, we also explored the possibilities of using the model to improve future data collection quality as a feedback loop.

## Introduction

There are more than 3600 known species of mosquitoes^1,2^. The majority are harmless to humans but a few dozen transmit diseases. Because of these species, mosquitoes are often referred to as the deadliest animals on Earth^3^. Different mosquito species, including the three main genera *Aedes*, *Anopheles*, and *Culex*, are able to transmit a variety of pathogens to humans (e.g. virus, parasites) when infected females feed on human hosts. Their ability to carry and spread disease to humans is responsible for hundreds of millions of infections and hundreds of thousands of deaths annually, imposing a massive global burden on society^4–11^.

The global transport of disease reservoirs and vectors, climate change, urban growth, ecosystem degradation, and habitat fragmentation are among the factors driving the recent emergence and re-emergence of mosquito-borne diseases (MBDs) worldwide^12^. In recent decades, for example, a large number of countries have reported their first dengue outbreaks, and the worldwide incidence of dengue has risen 30-fold in the past 50 years^12^. Dengue, along with chikungunya, Zika, yellow fever other diseases, are transmitted to humans by *Aedes (Stegomyia) albopictus* (Skuse, 1894) and *Aedes (Stegomyia) aegypti* (Linnaeus, 1762)^13^. The two species live together and share urban environments with more than half of the world’s population^14^.

In the absence of effective vaccine solutions for most MBDs^15^, attention has turned to the urgent strengthening of vector management^16^ as a fundamental approach to preventing disease and responding to outbreaks. To be successful, however, vector control programs require innovative tools for mosquito population surveillance and control, as well as much greater community participatory action and mobilization^17,18^.

Assessing the current distribution and dynamics of the targeted mosquito species is core to any control strategy, with efficiency depending on the early detection of invasive species and the effective monitoring of the spread and population dynamics of competent vectors in colonized territories (both invasive and native vectors). Obtaining authoritative data through traditional mosquito surveillance, e.g. by trapping adults, dipping for larvae, or ovitrapping, is costly, time-consuming, and labour-intensive, making this a barely scalable approach that is generally able to cover only small parts of any given country^19^. As a result, community-based approaches, in which citizens are provided the means to recognize, report, collect, and submit mosquito specimens are becoming increasingly popular, and receiving growing support from the scientific community^20,21^. This is the case of the citizen science platform Mosquito Alert (www.mosquitoalert.com/en).

**Figure 1:**
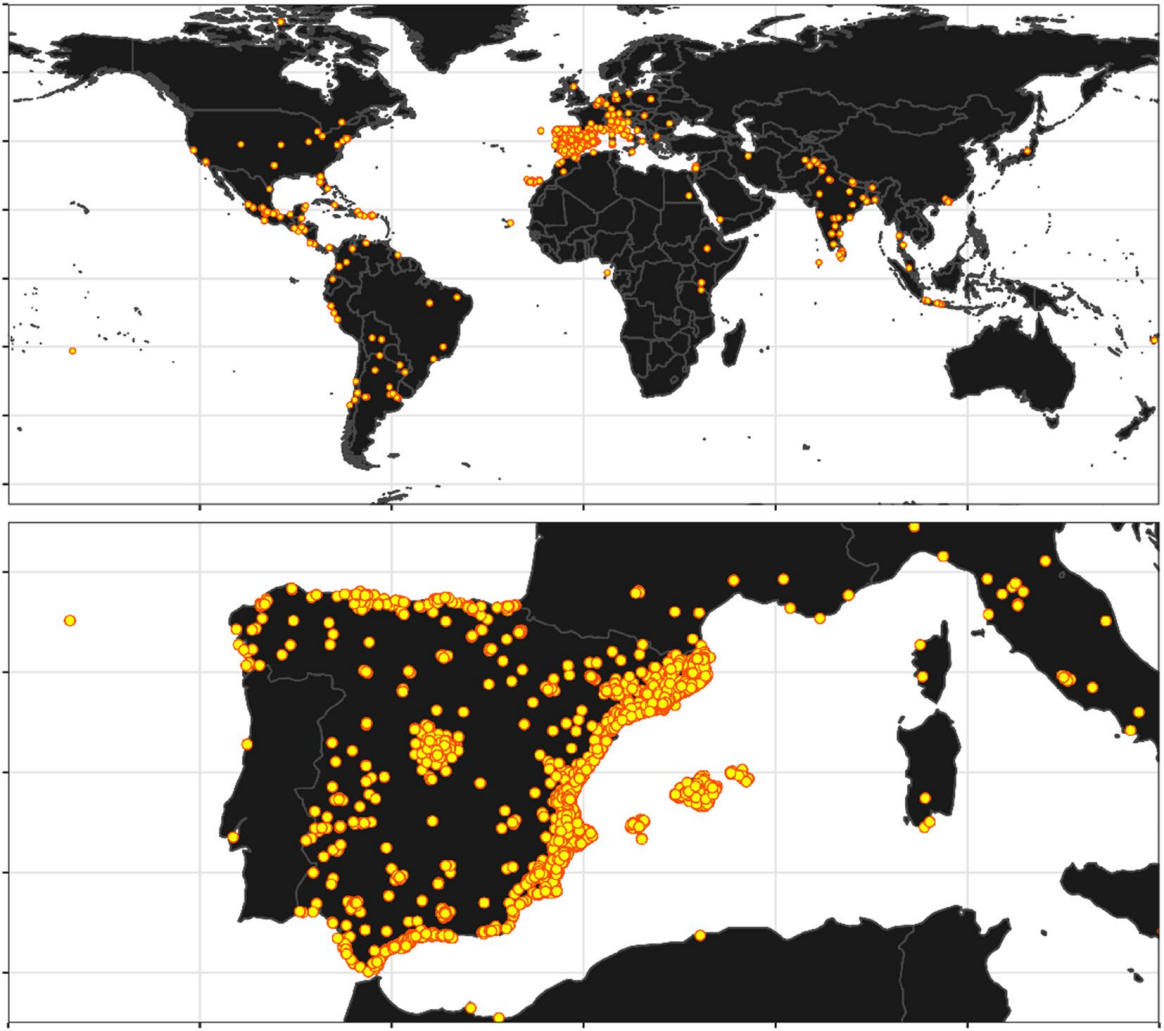
Geographical diversity of the submitted images based on the provided geolocation tags. It can be clearly seen that while the vast majority of the Mosquito Alert participants are based in Europe (particularly Spain), images were taken all over the world. The map was made with Natural Earth public domain map data (https://www.naturalearthdata.com/) using the rnaturalearth package version 0.1.0^24^ in R version 4.0.2^25^ (https://www.R-project.org/).

Mosquito Alert enables ordinary people to identify and report targeted disease-vector mosquitoes anywhere they encounter them worldwide (see Fig. 1). The system targeted tiger mosquitoes (*Ae. albopictus*) from 2014 and both tiger and yellow fever mosquitoes (*Ae. aegypti*) from 2016. At the end of 2020 it began targeting additional species as well (*Ae. japonicus*, *Ae. koreicus*, *Culex pipiens*), but the dataset analyzed in this article ends in 2019 when only tiger and yellow fever mosquitoes were being targeted.

The citizen scientists’ reports sent through Mosquito Alert are validated by entomologists and shared with control services and public health agencies. By also estimating participants’ sampling effort based on background geo-positioning, the system is able to make inferences about mosquito risk distribution of comparable quality to those generated from traditional ovitrap surveillance methods but in a more scalable and flexible manner^22^. A crucial aspect of this type of system is improving data quality, the so-called fitness of use^22,23^. One way this is done is by providing feedback to citizens through social media and community engagement when they fail to provide adequate pictures (blurred images, non-targeted species), and explaining tricks on how to catch mosquitoes and make good pictures with their smartphones^23^.

In the early 2010s a branch of machine learning models, convolutional neural networks (CNN) gained enormous attention and development. Since then, CNNs have reached and often outperformed human expert-level accuracy in various tasks. The recent rise of deep learning techniques is mainly due to the availability of large enough image databases and the increase in computing power. In this article, we demonstrate how CNNs can help to reduce the labour-intensive labeling process for the Mosquito Alert platform. As Mosquito Alert is an active ongoing project, there is a potentially great benefit to improving the data collection guidelines. The collected dataset provides a good opportunity to use CNNs for this purpose.

## Results

**Figure 2:**
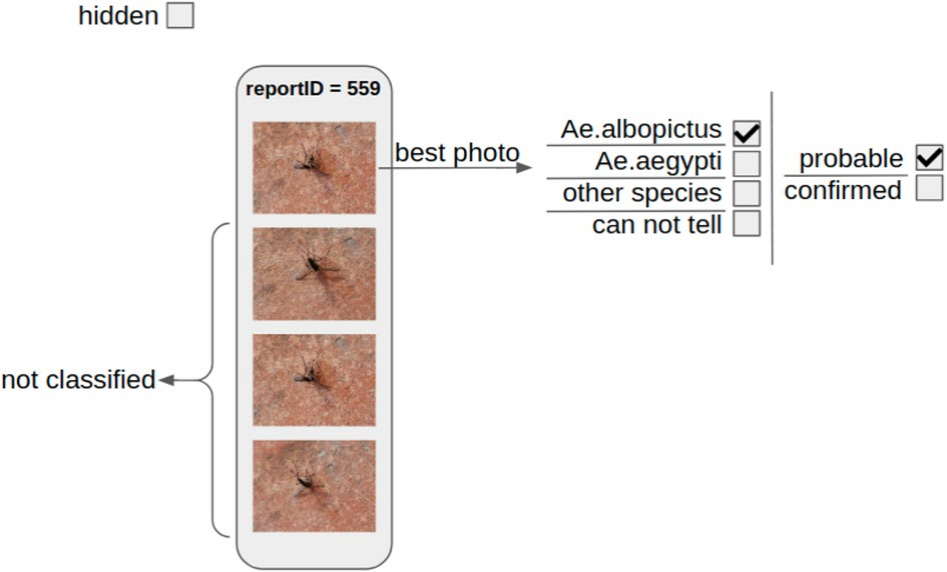
Schematic figure of the labeling process. Participants usually upload several images in a single report. The best photo is picked by the validator who first marks the harassing or non-appropriate photos as hidden. All the non-best photos are marked as not classified. In some rare events, two or three images are annotated from the same report. The mosquito images are classified into four different categories (*Aedes albopictus*, *Aedes aegypti*, other species or can not tell) and also the confidence of the label is marked as probable or confirmed. In this paper we excluded the not classified, the hidden and the can not tell images.

Between 2014 and 2019, 7686 citizen-made mosquito photos were labeled through Mosquito Alert by entomology experts, with labels indicating whether *Ae. albopictus* appear in the photos. The photos were included in reports that Mosquito Alert participants uploaded, and each report could contain several photos, see Fig. 2. The entomology experts usually labeled the best photo of the report, but sometimes they labeled two (420 times) or three (49 times) for a single report, meaning that the dataset consisted of 7168 reports. For 6699 reports, only one image was labeled by the experts; for 420 reports two were labeled; for 49 reports three were labeled. Although these reports usually contain several photos, only the ones with expert labels were used in the analysis, as cannot be assumed that all of the photos in a report would have been given the same label.

The main goals of Mosquito Alert during this 6 year period were to monitor *Ae. albopictus* spreading and provide early detection of *Ae. aegypti* in Spain. Although people participate in Mosquito Alert all over the world, the majority of the participants and the majority of the photos are in Spain (see Fig. 1). As *Ae. aegypti* has not been reported in Spain in recent times, most Mosquito Alert participants lived in areas where *Ae. aegypti* is not present, so most of the photos are of *Ae. albopictus*. For the detailed yearly distribution of the photos, see Table 1.

**Table 1:** The collected and expert validated dataset for the period 2014–2019.

	2014	2015	2016	2017	2018	2019	Total
Not *Aedes albopictus*	1	138	276	249	456	371	1491
*Aedes albopictus*	91	2156	901	960	1180	907	6195
Total	92	2294	1177	1209	1636	1278	7686

*Ae. albopictus* are clearly over-represented, which is expected because the system targeted only this species until 2016, and two species *Ae. albopictus* and *Ae. aegypti* from 2016 to 2019.

A popular deep learning model, ResNet50^26^ was trained and evaluated on the collected dataset with yearly cross-validation. ResNet50 was used because of its wide popularity and its proven classification power in various datasets. As presenting infinitesimal increments of the classification power is not a goal of this paper, we do not report various ImageNet state-of-the-art model performances. Yearly cross-validation was used to rule out any possibility of information leakage (possibility of a user submitting multiple reports for the same mosquito).

The trained model is not only capable of generating highly accurate predictions, but it can also ease the human annotator workload by auto-marking the images where the neural network is confident and more accurate, leaving more uncertain cases for the entomology experts. Moreover, while visualizing the erroneous predictions a few re-occurring patterns were identified, which can serve as a proposal for how to make images that can be best processed by the model.

Several aspects of the dataset were explored as follows.

### Classification

Since Mosquito Alert was centered around *Ae. albopictus* during the relevant time period (2014–2019), the collected dataset is biased towards this species (Table 1). We explored training classifiers on the Mosquito Alert dataset alone and also tied training on a balanced dataset, where 3896 negative samples were added from the IP102^27^ dataset of various non-mosquito insects as negative samples. From the IP102 dataset, images similar to mosquitoes, and images of striped insects were selected. Although the presented mosquito alert dataset is filtered to contain only mosquito images, in later use, non-mosquito images might be uploaded by the citizens. Training the CNN on a combination of mosquito and non-mosquito images can improve the model to make correct predictions, classifying non-tiger mosquitoes for those cases too. For testing, in each fold, only the Mosquito Alert dataset was used.

**Figure 3:**
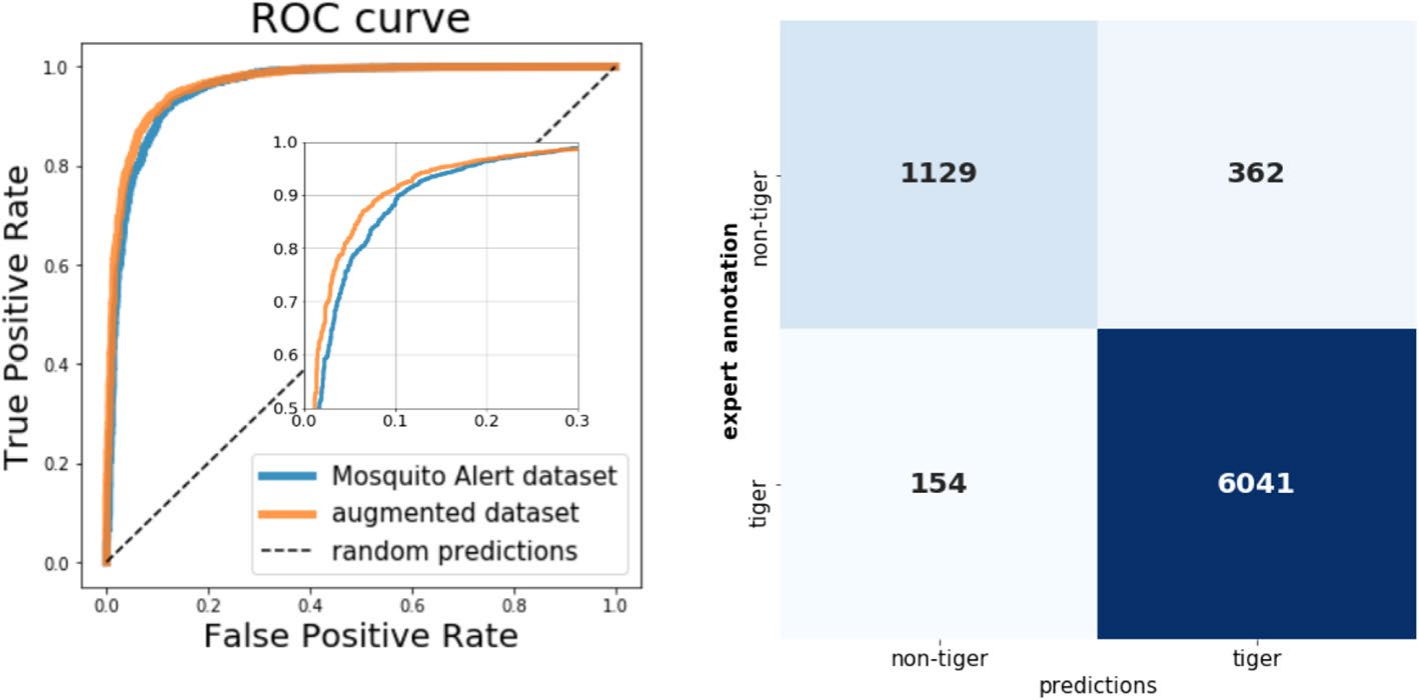
Left: ROC curve calculated on the prediction of the 7686 images in the Mosquito Alert dataset with yearly cross-validation. The blue line shows the case when only the Mosquito Alert dataset was used for training, the orange when the training dataset was balanced out with the addition of non-tiger mosquito insect images from the IP102 dataset. Also a zoom into the part of the ROC curve, where the two methods differ the most is highlighted. Right: the confusion matrix was calculated on the same predictions when only the Mosquito Alert dataset was used for training. For both, a positive label means tiger mosquito is present.

The trained classifiers achieved an extremely high area under the receiver operating characteristic curve (ROC AUC) score of 0.96 (see Fig. 3). The fact that the ROC AUC score for each fold was always over 0.95 proves the consistency of our classifier. Inspecting the confusion matrix shows us that the model tends to make more false positive predictions (assuming tiger mosquito is defined as the positive outcome) than false negatives, resulting in high sensitivity. The augmentation of the Mosquito Alert dataset with various insects from IP102 images to make it more balanced resulted in a slight performance boost and narrowed the gap between the number of false positive and false negative samples as expected, see Table 2.

**Table 2:** Yearly cross-validation results with using the Mosquito Alert dataset alone and its IP102 augmented version.

Used data	ROC AUC	TP	TN	FP	FN
Mosquito Alert dataset	0.9577	6041	1129	362	154
Augmented dataset	0.9663	5992	1190	301	203

The additional non-tiger mosquito insect images were used only to balance out the train dataset, they were not used in the validation dataset. The area under the receiver operating characteristic curve (ROC AUC), true positive (TP), true negative (TN), false positive (FP), false negative (FN) is shown.

### How to take a good picture?

Inspection of the weaknesses of a machine learning model is a fruitful way to gain a deeper understanding of the underlying problems and mechanisms. In our case, a careful review of the mispredicted images led us to useful insights into what makes a photo hard to classify for the deep learning model. On Fig. 8, a few selected examples are presented. Unlike humans, deep learning models rely more on textures than on shapes^28^. As a consequence, grid-like background patterns or striped objects may easily confuse the machine classifier. A larger rich training set can help to avoid these pitfalls, but we also have the option to advise the participants. If participants avoid confusing setups when taking photos, this can improve the accuracy of the automated classification. These guidelines can be added to the Mosquito Alert application to help participants make good images of mosquitoes.

- Do not use striped structure (e.g. mosquito net or fly-flap) as a background.
- Avoid complex backgrounds when possible. A few examples: patterned carpet, different nets, reflecting/shiny background, bumpy wallpaper.
- Use clear, white background (e.g. a sheet of plain paper is perfect if possible) or hold the mosquito with finger pads.
- Make sure that as much as possible the mosquito is in focus and covers a large area of the photo.

In general, it is desirable to have a clean white background with the mosquito centered, and with the image containing as little background as possible.

### Dataset size impact on model performance

Modern deep CNNs tend to generate better predictions when trained on larger datasets. In this experiment, we trained a ResNet50 model on 10–20–$\cdots$–90–100% of 6686 images and evaluated the model on the remaining 1000 images. The 1000 images were selected from the same year (2019) and all of them came from reports with only one photo. There were 709 tiger mosquitoes out of the 1000 test images. ROC AUC and accuracy were calculated with a 500 round bootstrapping of the 1000 test images.

**Figure 4:**
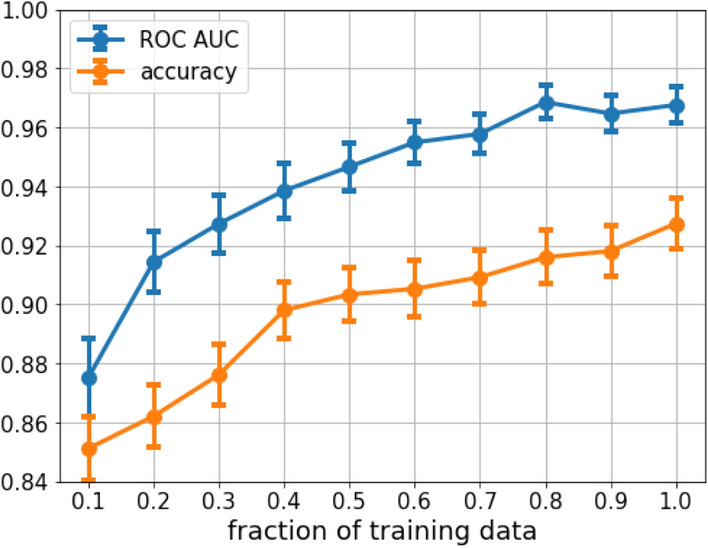
Training a ResNet50 model on a subsampled training dataset. The model was tested against the same 1000 test images for all the steps and statistics of the test metric was calculated with a 500 round bootstrapping. The curve proves the diversity of the Mosquito Alert dataset and also suggests that in the future when the dataset will be even larger, the classification performance will increase.

The mean and the standard deviation of the 500 rounds are shown in Fig. 4 for each training data size. From the figure, we can conclude that the predictive power of the model increases as more data are used. The shape of the curves also suggests that the dataset did not reach its plateau. In the upcoming years, as the dataset size increases, ROC AUC and accuracy enhancement is expected.

### On measuring image quality

Through the examined period, Mosquito Alert outreach was promoting a mosquito-targeted data collection strategy. Participants were expected to report two mosquito species (*Ae. aegypti* and *Ae. albopictus*). By defining these species as positive samples and all the other potential species of mosquito as negative, the submission decision by participants becomes a binary classification problem. In the majority of cases, when participants submit an image we should expect them to think of having a positive sample. Later, based on entomological expert validation, the true label for the image was obtained.

The main goal of such a surveillance system is to keep the sensitivity of the users as high as possible while keeping their specificity at an acceptable level. Therefore, measuring the sensitivity and specificity of the users would be a plausible quality measure. Unfortunately, there is no available information regarding the non-submitted mosquitoes (the true negative and false negative ones), meaning it is impossible to measure sensitivity. The specificity can be measured only in a special case, when there are no false positive images submitted by the user, resulting in a specificity of 1. Based on the latter argument, focusing on metrics derived from the ratio of the submitted tiger mosquito images vs. all submitted images is not meaningful. Instead, the quality can be measured by the usefulness of the photos from the viewpoint of the expert validator or a CNN, as presented in the next chapter.

### Quality evolution of the images through time and space

The Mosquito Alert dataset is a unique collection of mosquito images, because, among other things, it is built from 5 consecutive years (not counting 2014, where less than 100 reports were submitted) and it also provides geolocation tags. This uniqueness of the dataset provides potential identification of time and spatial evolution and dependence of the citizen-based mosquito image quality. To explore such an evolution, we performed two different experiments. Geolocation tags were converted to country, region, and city-level information via the geopy Python package. It was found, that the vast majority (95% of all) of the reports were coming from Spain so we performed the analysis only for the Spanish data.

**Figure 5:**
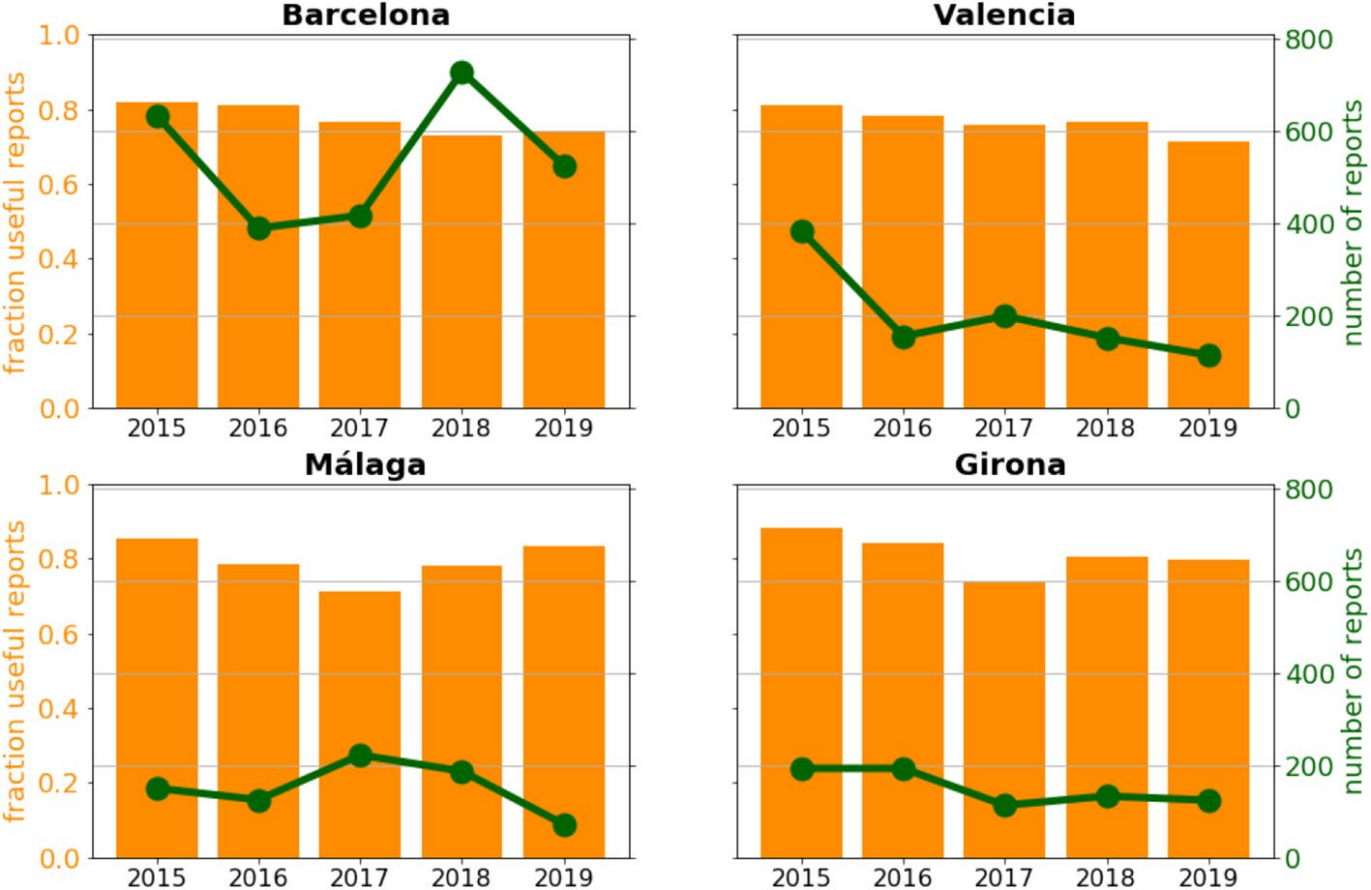
Number of submitted reports and the fraction of their ratio where the entomology expert annotator could tell if tiger mosquito was presented on the photo or not. The charts are shown for the four cities, where Mosquito Alert was the most popular.

First, we explored the fraction of the photos, where the entomology expert marked “can not tell”, because the photo was not descriptive enough to decide which species were presented. Figure 5 shows the ratio of the useful mosquito reports, when mosquito decision was possible, compared to all the mosquito reports. The chart shows the above-mentioned ratio for four Spanish cities, which have the most reports submitted (the same information is showed on Supplementary Fig. S1 as a heatmap over Spain). The Mann–Kendall test on the fraction of useful reports shows p-values of 0.09, 0.09, 0.81, 0.22 for Barcelona, Valencia, Málaga, and Girona, which does not justify the presence of a significant trend in image quality, although any conclusions drawn from five data points must be handled with a pinch of salt. It does not mean anything about the individual participants’ quality progression, because Mosquito Alert is highly open and dynamic, and active participants can constantly change. Of note, through these years, the tiger mosquitoes have widely spread from the east coast to the southern and western regions of Spain^29^. New (and naive) citizen scientists living in the newly colonized regions have been systematically called to action and participation, thus, limiting the overall learning rate of the Mosquito Alert participants’ population. Our results suggest, that either a dynamic balance exists between naive and experienced participants over the period of data recollection, or mosquito photographing skills are independent of the user experience level. The expectation would be that as the population in Spain became more aware of the presence of tiger mosquitoes and their associated public health risks, the system should experience an increase in the useful report ratio, at least for tiger mosquitoes, and most tiger mosquito photos maybe classified automatically.

**Figure 6:**
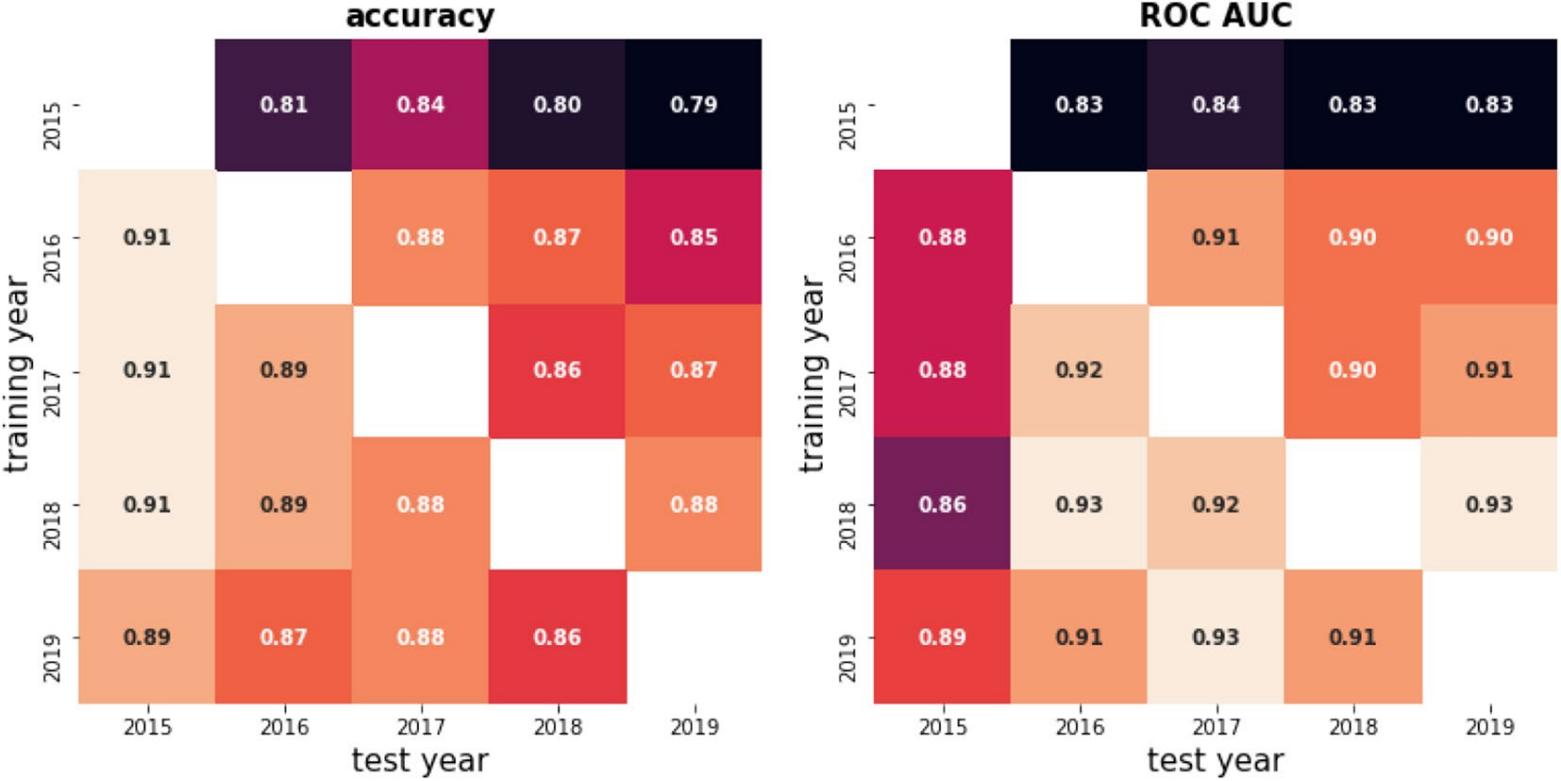
1000 random samples were selected for each years data. Separated ResNet50 models were trained on each of the years and each model was tested on the rest of the years data. Metrics were calculated with a 500 round random sampling with replacement from the test data. Left: mean of the 500 round bootstrapped accuracy calculations. Right: mean of the 500 round bootstrapped ROC AUC calculations.

Second, we subsampled randomly 1000 images from all years between 2015 and 2019. Then we trained a different ResNet50 on data from the different years and generated predictions for the rest of the data, for each year separately. This way we can explore if data from any year is a “better training material” than the others. The results see Fig. 6, shows that 2015 is the worst training material, providing 0.83–0.84 ROC AUC score for the test period, while the rest (period 2016–2019) is similar, ROC AUC varies between 0.90 and 0.93. The reason why the 2015 data found to be the least favourable for training is its class imbalance, meaning that data from 2015 is extremely biased towards tiger mosquitoes (94%), so when training on 2015 data, the model does not see enough non-tiger mosquito samples, while for the other years lower class imbalance was found (70–80%), see Table 1. In general, machine learning models for classification require a substantial amount of examples for each possible class, in our case tiger and non-tiger mosquitoes, therefore worse performance is expected when training on the 2015 data.

Other than the varying class imbalance, we can conclude that the Mosquito Alert dataset quality is consistent, we did not find any concerning difference between training and testing our model for any of the 2016–2017–2018–2019 data pairs.

### Pre-filtering the images before expert validation

Generating human annotations for an image classification task is a labour-intensive and expensive part of any project especially if the annotation requires expert knowledge. Therefore, having a model that generates accurate predictions for a well-defined subset of the data saves a lot of time and cost. We assume that the trained classifier is more accurate when the prediction probability is whether high or low and more inaccurate when it is close to 0.5. With this assumption in mind one can tune the $p_{low}$ and $p_{high}$ probabilities, in a way that images with a prediction probability $p_{low}< p < p_{high}$ are discarded and sent to human validation.

**Figure 7:**
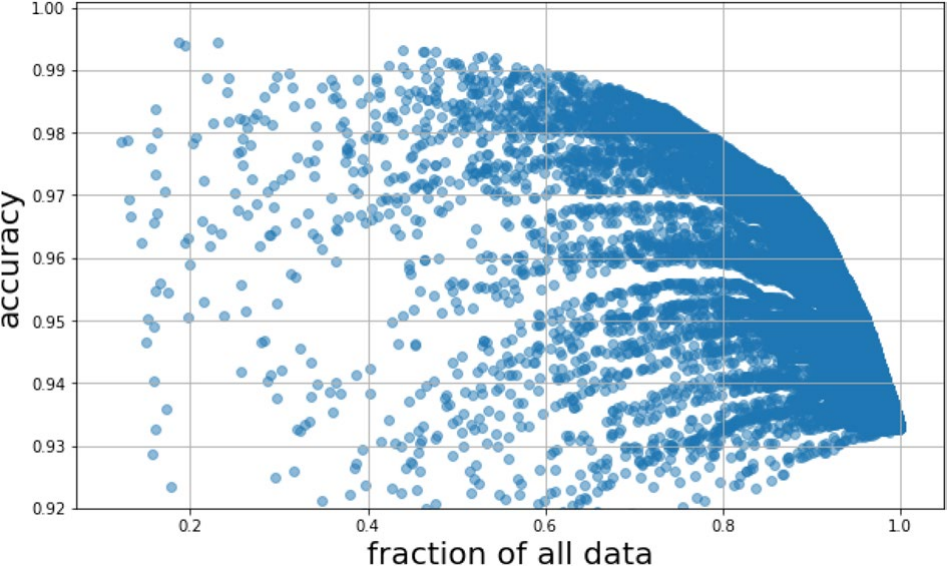
Randomly selecting 100,000 $p_{low}$ and $p_{high}$ thresholds on the predictions which were created via yearly cross-validation. Each time only samples were kept where the predicted probability were out of the $[p_{low};p_{high}]$ interval. Each point shows the kept data fraction and the prediction accuracy. Varying the lower and upper predicted probability almost 98% of the images are correctly predicted while keeping 80% of all the images.

Varying $p_{low}$ and $p_{high}$ provides a trade-off between prediction accuracy and the portion of images sent to human validation. Based on Fig. 7 sending 20% of the images to human validation while having an almost 98% accurate prediction for 80% of the dataset is a fruitful way to combine human labour-power and machine learning together.

## Discussion

Citizen science methodologies have been sharpened in the last decade, leading to greater acceptance among the scientific community, which was initially concerned about data quality and sampling biases. Successful citizen science projects can be found across a wide range of disciplines, from the life sciences, where they help assess biodiversity at a global scale (e.g. iNaturalist, iSpot), to astronomy, where they solve the morphological classification of galaxies (e.g. Galaxy Zoo)^30^, to many more. For example, iNaturalist enables citizen scientists to upload pictures of any species, powering a big data approach to biodiversity quantification and worldwide conservation. In iNaturalist, automated species identification is now a key feature and an active research question^31^.

The citizen science approach is an excellent way to collect information (whether raw data or labeling) on any topic when automation is not yet possible. The collected information can be orders of magnitudes larger and much more diverse compared to traditional data collection methods. Despite the fact that data quality is less guaranteed, it can be adjusted to specific needs by means of statistical methods and community engagement techniques. Next-generation citizen science methodologies should combine expert-wise classification with machine learning classifiers. The bottleneck is to generate good enough supervised datasets for the machine learning model to be able to learn from them.

Many states around the world are required to implement a nationwide plan for disease vector mosquito surveillance. National plans for mosquito surveillance often follow guidelines produced at institutions like the European Centre for Disease Prevention and Control (ECDC)^32^ or the World Health Organization (WHO)^16^. The WHO has been pushing for programs that bring together multiple vector control strategies in cost-effective, flexible, and sustainable ways. Recently, these plans have been augmented or complemented with citizen science^20^. As a next step, citizen science observation maps need to be standardized and coupled with disease data for the best utilization.

Citizen science has been used to track insects more generally in the Monitoring of Insects with Public Participation (MIPP)^33^ project, and other specific groups like butterflies in the Catalan Butterfly Monitoring Scheme^34^. In the context of public health, citizen science has also been implemented in crisis scenarios, as for example in the Safecast project^35^ in the Fukushima earthquake in 2011 that followed the nuclear energy catastrophe. Indeed, citizen science based surveillance of vector mosquitoes may be operative for both public health preparedness and crisis scenarios (epidemic bursts). Mosquito Alert, for example, is used as a surveillance system at the national scale (note the detection with this system of a new mosquito vector in 2018 in Spain^36^), and also as a control system in the city of Barcelona^23,37^, where the public health agency of Barcelona (ASPB) treats the reporting activity of citizens as an incidence map that it uses in combination with available data to plan weekly cost-effective control measures over the whole mosquito season. In addition, in the case of epidemic bursts, novel technologies in the hands of prepared citizens can easily spread the word on risk minimization behaviours, increase social awareness, and produce a flexible and scalable data collection system (mosquito pictures, breeding sites or biting patterns) for decision-making and management. In line with the next-decade’s vector control road-map, and in most of the above-mentioned context, it is crucial to combine the benefits of community-driven citizen science with cutting-edge machine learning tools from the ongoing revolution in big data. Not only can they these tools improve scalability by reducing the number of images that require an expert human eye, but these systems will also be refined. Some limits exist in that not all taxonomic characters are observable without a microscope and some species are too similar. Nonetheless, the improvement in machine learning methods, together with the refinement of future human-in-the-loop interactions, and the inclusion of other big data sources (environment, mosquito behaviour, participant activity and profiling) might make machines better at discriminating species than an untrained human eye, and in some cases better than a trained eye. The presented work is the first step toward this goal and shows that deep-learning tools can be implemented to leverage and improve the scalability of community, and smartphone-based mosquito reporting.

Convolutional neural networks were used to classify mosquito species by images in the literature. While these research reports exceptionally high accuracy^38–41^, their method is usually based on consistently high-quality images, which are relatively rare in systems where images are taken by non-experts randomly selected. Others have created an automated way of taking photos of live mosquitoes and trained CNNs on the collected data^42,43^. This is a promising way to achieve automated mosquito surveillance.

Nonetheless, one can already acknowledge some strong methodological limits when using mosquito images. Among the few (order of dozens) disease-carrying mosquitoes of concern, not all of them can be identified on the basis of a photograph. Key taxonomical characters are often hidden from the human eye and require animal dissection and material preparation for observation through a binocular lens or microscope. In this context it is important to mention that there has been substantial effort put into automated mosquito classification from other sourced data, that is, targeting the problem from other angles. As an example, some works have attempted to predict the presence of a mosquito or a given mosquito species from its buzz^44–47^. The wingbeat sound can be recorded via a standard microphone, or else recording the air pressure change or the movement of the wing that is converted into soundtracks by an optical sensor. While recording the buzz of mosquitoes attracted by lures on the field might be a viable option, it is much more difficult in urban areas due to noise pollution. Wing interference patterns (WIP) are also a promising data source to classify mosquitoes^48^, but its usage by citizens is complicated because it requires much more practice and effort to manipulate the specimen to create high-quality WIP images compared to a traditional photo.

The presented work focuses on *Ae. albopictus* because that is the main targeted species for the period 2014–2019 in the Mosquito Alert project, but the targeted species were extended recently^49^. Machine learning classification of other mosquito species could be done but may require some time, as citizens need to learn how to make good pictures of them, and also large enough sample size is required for training a model. One of the advantages of the Mosquito Alert citizen science program is the ability to be able to communicate with citizens directly (through smartphone notifications) and indirectly (through the web or social media). Part of this communication has to do with providing feedback and tricks in order to improve image quality, highlighting what body parts of the mosquito are the key from a taxonomic perspective. In this regard, both the improvement of deep learning techniques and expertise by citizens may facilitate mosquito classification.

In the future, it is likely that different sources of data could be combined in order to improve automated mosquito classification together with technical and deep learning methods refinement.

## Methods

### Data collection

The photos were made and submitted by participants of the Mosquito Alert citizen science platform. Through the Mosquito Alert smartphone application, participants can upload several images within a single report belonging to the same mosquito. The application is freely available both in the App Store and Google Play. While creating the report the app obtains high-resolution location information from the mobile phone sensors and incorporates this into the report too. The photos are stored at medium resolution, usually varies between 150 and 500 pixels in both dimensions.

**Figure 8:**
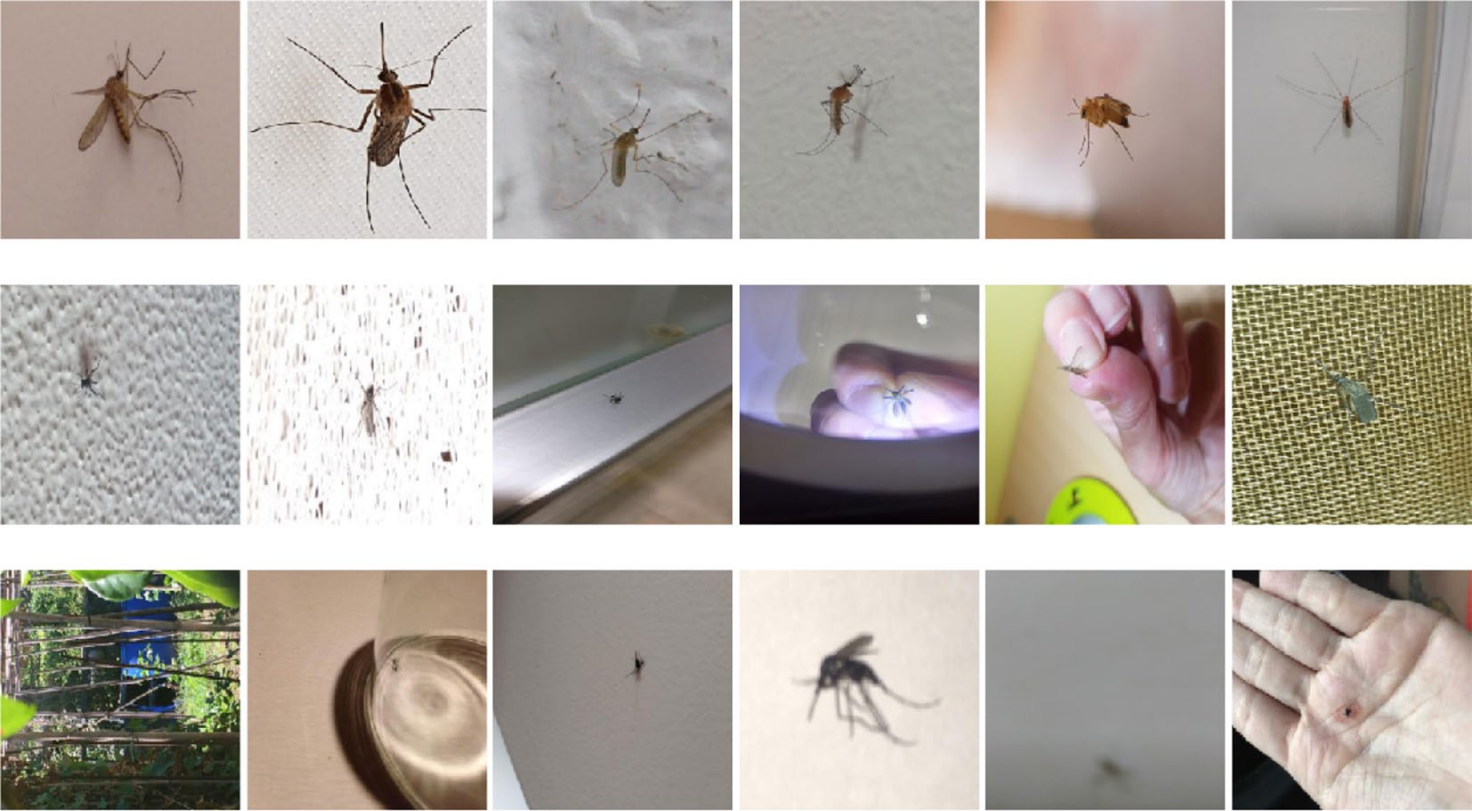
Top row: correctly classified images, where the model was confident, thus assigned close to 1 probability to the correct category. Middle row: examples from the images which were mistakenly predicted by the CNN model. Bottom row: selected examples where the entomology expert could not tell if the presented mosquito is a tiger mosquito or not. The images were resized to have the same aspect ratio for visualization purposes.

### Expert validation

Uploaded mosquito images are classified by entomology experts. Each image is validated by one expert and the mosquito images are classified into one of the following categories:

- *Ae.albopictus*
- *Ae.aegypti*
- other species
- can not tell

For each category, the validator can mark whether the prediction is confirmed or just probable. In the application photos of breeding sites are also collected, but we do not use that data in the presented research. Figure 2 shows the labeling process in a schematic view.

### Data filtering

The inappropriate images, marked as hidden, see Fig. 2 were excluded from this study. Also, we did not use the not classified and the can not tell mosquito images. From the mosquito and breeding site images, only the mosquito images were used in the research.

### Collected dataset

The collected dataset is publicly available, the link is provided in the Additional information section.

All non-mosquito images were discarded, but both confirmed and probable labels were kept resulting in a total of 7686 images, see Table 1. As the dataset is collected through a citizen science project a wide variety of backgrounds, blurriness, zoom level, and resolution is experienced as it can be seen on Fig. 8.

### Training the neural network

An ImageNet pre-trained ResNet50^26^ model was trained with yearly cross-validation on the dataset via the FastAI API^50^. ResNet50 was selected because this well-popular architecture performs well in general image recognition tasks, and because of its popularity, ResNet50 provides an easy to re-implement benchmark for our work.

The training was done with fit one cycle policy for ten epochs, using a maximum learning rate of $10^{-5}$ for the first five epochs and $5 \times 10^{-5}$ for the second five epochs using categorical cross-entropy loss. Also, in the first five epochs only the fully connected last layer was trained, while in the last five epochs all the layers were fine-tuned. Mini-batch size of 32 was used.

First, all images were resized to $256 \times 256$ pixels. During training various image augmentation techniques were applied, such as horizontal flipping, random rotation of the image, zooming to the image, changing the lighting, and perspective warping. The program code that was used for training is accessible at http://github.com/patbaa/mosquitoalert_classification.

The training process with ten epochs took 10–15 min on an Nvidia GTX 1070 GPU.

### Evaluation

Evaluation of the deep learning model was performed via yearly cross-validation in order to exclude the information leakage between images when several images were taken of a single mosquito.

When the prediction was generated for all the images, the ROC AUC score was calculated. ROC AUC was also calculated for all folds separately in order to ensure the consistency of the method. Additionally, the confusion matrix was created, see Fig. 3, from that all the usual metrics (sensitivity, specificity, accuracy, F1 score) can be easily calculated.

During the evaluation, no test time augmentation was used.

### Metrics

#### ROC AUC

Area under the receiver operating characteristic curve, which means plotting the $\frac{TP}{TP + FN}$ on the y-axis while $\frac{FP}{TN + FP}$ on the x-axis for various probability cutoff thresholds, where TP is the number of the true positive samples, TN, FP, FN are the false negatives, false positives, and false negatives.

#### Accuracy

The fraction of the correctly predicted samples. $\frac{TP + TN}{TP + TN + FP + FN}$ with the notation used for the ROC AUC.

#### Sensitivity

$\frac{TP}{TP + FN}$, using the notation introduced in the “ROC AUC” section above.

#### Specificity

$\frac{TN}{TN + FP}$, using the notation introduced in the “ROC AUC” section above.

#### Bootstrappig

In some experiments bootstrapping was used. It was performed by resampling the whole test set with replacement and calculating the given score for that resampling. The resampling was performed 500 times, resulting in 500 estimates of the metric.

## Supplementary information

Supplementary Figure S1.

## Data Availability

The collected images are accessible at http://www.mosquitoalert.com/en/mosquito-images-data-base/.

## Code availability

The code is accessible at http://github.com/patbaa/mosquitoalert_classification. The additionally used IP102^27^ insect images are available from the authors of the IP102 paper upon request.
