# Supplementary material for: Deep learning identification for citizen science surveillance of tiger mosquitoes
Source: Sci Rep. 2021 Feb 25;11:4718. doi: 10.1038/s41598-021-83657-4 (PMC7907246; doi:10.1038/s41598-021-83657-4)
Supplement: Supplementary file 1 — Supplementary Figure S1. [file 41598_2021_83657_MOESM1_ESM.pdf]

Deep learning identification for citizen science surveillance of tiger mosquitoes

Balint Armin Pataki[1,*], **Joan Garriga**[2], **Roger Eritja**[3], **John RB Palmer**[4], **Frederic Bartumeus**[2,3,5], **and Istvan Csabai**[1]

[1]Department of Physics of Complex System, ELTE Eötvös Loránd University, Budapest, Hungary
[*]Corresponding author's email address: patbaa@caesar.elte.hu
[2]Centre d'Estudis Avançats de Blanes (CEAB-CSIC), 17300 Girona, Spain.
[3]CREAF, 08193 Cerdanyola del Vallés, Spain.
[4]Universitat Pompeu Fabra, 08005 Barcelona, Spain.
[5]Institució Catalana de Recerca i Estudis Avançats (ICREA), 08010 Barcelona, Spain.

Supplementary materials

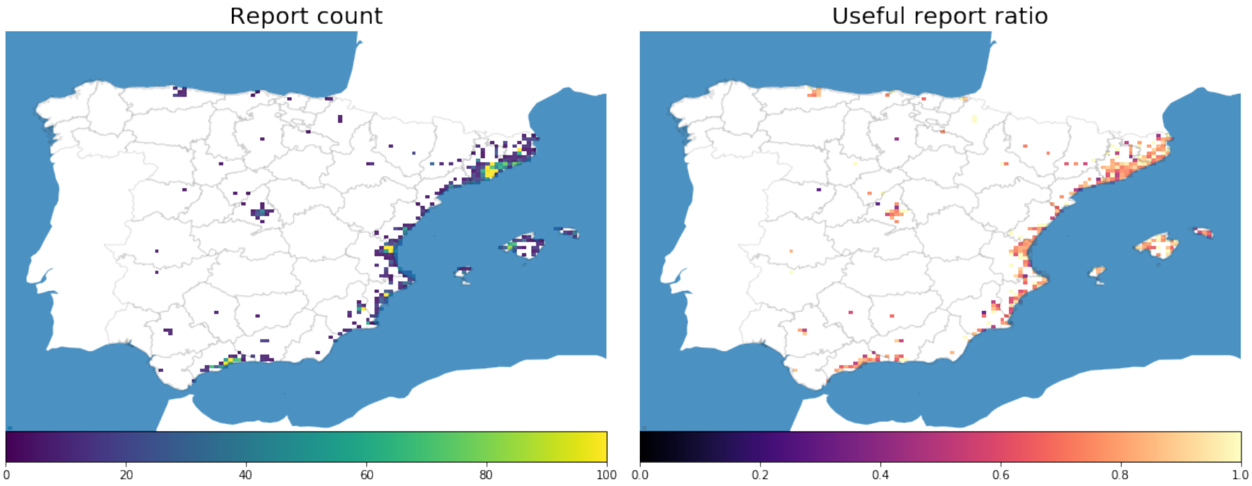

Figure S1. Image quality visualization for data coming from Spain. The submitted reports were aggregated on a grid (each cell covers roughly 10 km x 10 km). Only gridpoints with more than 3 submitted reports are shown. **Left:** the number of submitted reports as a heatmap. The colormap was thresholded at 100 for aesthetic purposes. Most of the reports are submitted by citizens from the eastern and southern regions of Spain, such as from Barcelona, Valencia, Málaga, and Girona. **Right:** the ratio between the useful reports and all reports is shown. A report is considered useful when the human annotator could decide if the targeted mosquito species, *tiger mosquito*, was presented on the photo or not. Almost all locations have scoring above 0.5 and in particular high scoring (> 0.9) are scattered all over Spain and not clustered in a given region. This is true even if most of the grid locations showed less than 20 reports. So despite having few reports (not being able to have a clear quantitative picture of the data statistics) what is remarkable is that there are high-quality reports among them. These qualitative results do not seem to be changing as we change grid size. The analysis performed on the most active cities in terms of participation and during the period analyzed, see figure in the main text, is also coherent with these results: a similar proportion of high-quality reports are observed in all of the cities. The presence of high-quality reports scattered all over Spain, and including regions with a small number of reports, suggests that despite the outreach and communication strategy of Mosquito Alert is shaped for a broad audience, the initial project penetration in a new territory may come from skilled or relatively trained citizen rather than from a bulk of naive citizen. Clearly, as the initiative becomes more consolidated in a territory, relatively more inexperienced (naive) citizen are added on. The complex dynamics of consolidated and new territories of participation across these years of Mosquito Alert implementation in Spain may also explain the lack of a clear increase of image quality across time.
